# Scope review of self-management intervention for arteriovenous fistula in hemodialysis patients

**DOI:** 10.3389/fpubh.2026.1859375

**Published:** 2026-09-15

**Authors:** Yi Pan, Huimin Dong, Jie Yu, Xiaoping Wu

**Affiliations:** Department of Nursing, The Fourth Affiliated Hospital of School of Medicine, and International School of Medicine, International Institutes of Medicine, Zhejiang University, Yiwu, China

**Keywords:** arteriovenous fistula, hemodialysis, intervention studies, scope review, self-management

## Abstract

**Objectives:**

To map and characterize the available evidence on self-management interventions for arteriovenous fistulas in hemodialysis patients, identifying the types of interventions, their characteristics, and existing knowledge gaps.

**Methods:**

This review followed JBI methodology for scoping reviews and reported in line with the Preferred Reporting Items for Systematic Reviews and Meta-Analyses extension for Scoping Reviews (PRISMA-ScR). PubMed, Web of Science, The Cochrane Library, Embase, CINAHL, Scopus, CNKI, Wanfang Database, VIP database and China Biology Medicine Disc were systematically searched. To identify intervention studies focusing on the self-management of arteriovenous fistula in hemodialysis patients. The search timeframe extended from the inception of each database until December 2025. The language types of the included literatures were Chinese and English. Two independent researchers screened the literature, extracted relevant data, and conducted analyses on the included studies.

**Results:**

A total of 2073 articles were retrieved, and 15 studies were included. On the theoretical foundation, seven studies explicitly adopted theoretical foundation to guide intervention design, including The Transtheoretical Model of Behavior Change, Orem’s General Nursing Theory, The Contractual Management Model, The PRECEDE-PROCEED Model, Root Cause Analysis, and Ebbinghaus Forgetting Curve. In terms of intervention design and implementation, most of the programs were developed jointly by clinicians and nurses, and nurses were the main implementors. The intervention contents mainly included Arteriovenous fistula related knowledge, daily assessment and nursing, precautions and complication identification. Some studies also involved diet, exercise, medication and hemodialysis related management. The main form of intervention was combined strategy, and individualized face-to-face education, multimodal health education, mobile health management and peer support were common. In the included studies, the total duration of the intervention ranged from 2 to 8 weeks, the frequency ranged from 1 to 8 sessions, and the duration of a single intervention ranged from 15 min to 60 min. In terms of outcome evaluation, most studies used self-management ability as the primary outcome index, 7 studies reported complication outcomes, 4 studies evaluated psychological status, and 3 studies evaluated quality of life, while there were relatively few studies on physiological and biochemical indicators and physical function.

**Conclusion:**

Studies on self-management intervention of arteriovenous fistula in hemodialysis patients have shown certain effects, but it is still in the development stage. This review sorted out the characteristics and shortcomings of the existing evidence, so as to provide ideas for follow-up research design and clinical practice improvement, in order to eventually improve the self-management ability and long-term outcomes of arteriovenous fistula in hemodialysis patients. Playing the role of multidisciplinary team and using mobile applications to build a multi-level and personalized self-management intervention plan may be a feasible direction to improve the intervention effect.

**Systematic review registration:**

The protocol is registered on OSF with the unique identifier: https://doi.org/10.17605/OSF.IO/YRBTZ, publicly available at: https://osf.io/26eb4.

## 1 Introduction

After chronic kidney disease (CKD) progresses to end-stage renal disease (ESRD), patients need to rely on renal replacement therapy to maintain life. Hemodialysis is one of the most commonly used renal replacement therapy in clinical practice. And stable and effective vascular access is ensure the smooth implementation and dialysis adequacy of hemodialysis (1).

Currently, commonly used vascular access includes central venous catheter (CVC), arteriovenous graft (AVG), and arteriovenous fistula (AVF) (2). Among them, AVF is regarded as the preferred first-line vascular access because of its high patency rate, low risk of infection, long service life, low mortality and less need for subsequent intervention. However, complications such as stenosis, thrombosis, infection, bleeding and arteriovenous aneurysm may occur in the long-term use of arteriovenous fistula, which will affect its function and service life (3). One study showed that 47.5% of patients required further intervention to maintain patency of arteriovenous fistulas or treat complications (4). Therefore, improving the ability of hemodialysis patients to identify, prevent and cope with arteriovenous fistula abnormalities is of great significance to reduce the occurrence of complications and prolong the service life of AVF.

The long-term maintenance of AVF not only depends on the professional evaluation of medical staff during dialysis, but also needs patients to actively undertake the tasks of fistula side limb protection, tremor and bruit detection, and functional exercise in daily life. Previous studies have shown that the self-management level of AVF in hemodialysis patients is closely related to the occurrence of complications (5). A cross-sectional study showed that about 69.9% of hemodialysis patients still had a low or moderate level of AVF self-management (6). Another study showed that 94.9% of hemodialysis patients had mastered self-care knowledge, but did not strictly implement self-care behavior in actual life (7). This suggests that there is still a certain “knowledge-practice disconnect” phenomenon in the management of AVF. Patients’ demographic characteristics, cultural status, health literacy and access to continuing education and other factors may affect their self-management behavior (7). Existing studies have attempted to adopt multi-dimensional integrated intervention strategies. For example, a comprehensive intervention study on the prevention of AVF stenosis combined upper limb functional exercise, dietary guidance and self-management education, and the results showed that the upper limb muscle strength and AVF blood flow velocity of the patients were improved, the blood phosphorus level was decreased, and the self-efficacy score was improved after the intervention (8). In addition, a quasi-experimental study conducted by Sousa et al. (9) combined theoretical teaching with AVF self-care skills training to conduct a structured self-care intervention for 89 hemodialysis patients. The results showed that the overall frequency of self-care behavior in the intervention group increased from 79.2 to 91.4%, and the dimensions of symptom and signs management and complication prevention also showed an improvement trend. The above studies show that the self-management intervention of AVF has gradually expanded from simple knowledge education to behavior training, functional exercise, diet management and continuous support.

However, the existing studies vary greatly in terms of intervention makers and implementors, theoretical foundation, intervention content, intervention form, overall intervention period, frequency and duration of a single intervention, and the selection of outcome indicators and measurement tools are also lack of consistency. Most studies have focused on proximal outcomes such as knowledge level, self-management behavior, and self-efficacy, while relatively insufficient attention has been paid to long-term AVF function, complications, quality of life, and other patient-reported outcomes. Therefore, it is difficult to directly compare the available evidence, and it is difficult to define the core composition of different intervention programs and their applicable characteristics.

Thus, this study used scope review method to systematically retrieve and sort out related research on self-management intervention of AVF in hemodialysis patients, aiming to describe the overall characteristics of intervention research in this field, and summarize the theoretical foundation, intervention makers and practitioners, intervention content, intervention form, overall intervention period, frequency and duration of single intervention, and outcome indicators. To identify the evidence gaps and methodological limitations of existing studies, so as to provide evidence-based reference for the subsequent construction of standardized and localized AVF self-management intervention programs.

## 2 Methods

### 2.1 Study design

This scoping review follows Arksey and O’Malley framework (10) and guided by the JBI Manual (11) for Evidence Synthesis. The report adhered to the Preferred Reporting Items for Systematic Reviews and Meta- Analyses extension for Scoping Reviews (PRISMA-ScR) checklist (12). The protocol was registered on the Open Science Framework (OSF) website^1^. The registration number is https://doi.org/10.17605/OSF.IO/YRBTZ.

### 2.2 Identified the research questions

The study questions were based on the JBI’s Population-Concept-situation principle (PCC) (11). Population: maintenance hemodialysis patients with vascular access to an arteriovenous fistula, aged over 18 years. Concept: hemodialysis patients with arteriovenous fistula in self-management intervention. Context: studies in any environment, including hospitals, community or family. Based on this, the main research question of this review are as follows: arteriovenous fistula in hemodialysis patients with self-management intervention research plan, including: theoretical foundation, intervention makers and implementers, intervention content and form of intervention, the overall intervention period, frequency and duration of single intervention, outcome indicators. The secondary research question was: What are the types of outcome indicators for self-management interventions of AVF in hemodialysis patients?

### 2.3 Inclusion and exclusion criteria

Inclusion criteria:(1) the articles published in Chinese or English; (2) The subjects were maintenance hemodialysis patients with arteriovenous fistula, older than 18 years old; (3) intervention studies on improving the self-management ability of arteriovenous fistula in hemodialysis patients; (4) Only original studies with intervention design were considered, including randomized controlled study design, before-after controlled study and non-randomized controlled study.

Exclusion criteria included: (1) dissertations, conference abstracts, and research protocols; (2) non-Chinese and non-English literature.

### 2.4 Search strategy

Relevant studies were searched in PubMed, Web of Science (WOS), The Cochrane Library, Embase, CINAHL, Scopus, China National Knowledge Infrastructure (CNKI), Wanfang Database, VIP Database and China Biology Medicine Disc (CBM). The literature search for this study focused on hemodialysis, AVF, and self-management, with the search strategy tailored to meet the specific requirements of each database. The literature search was limited to Chinese and English through the database language filter. The search timeframe extended from the inception of each database until December 2025. Figure 1 details the specific search strategy and the number of articles retrieved.

**Figure 1:**
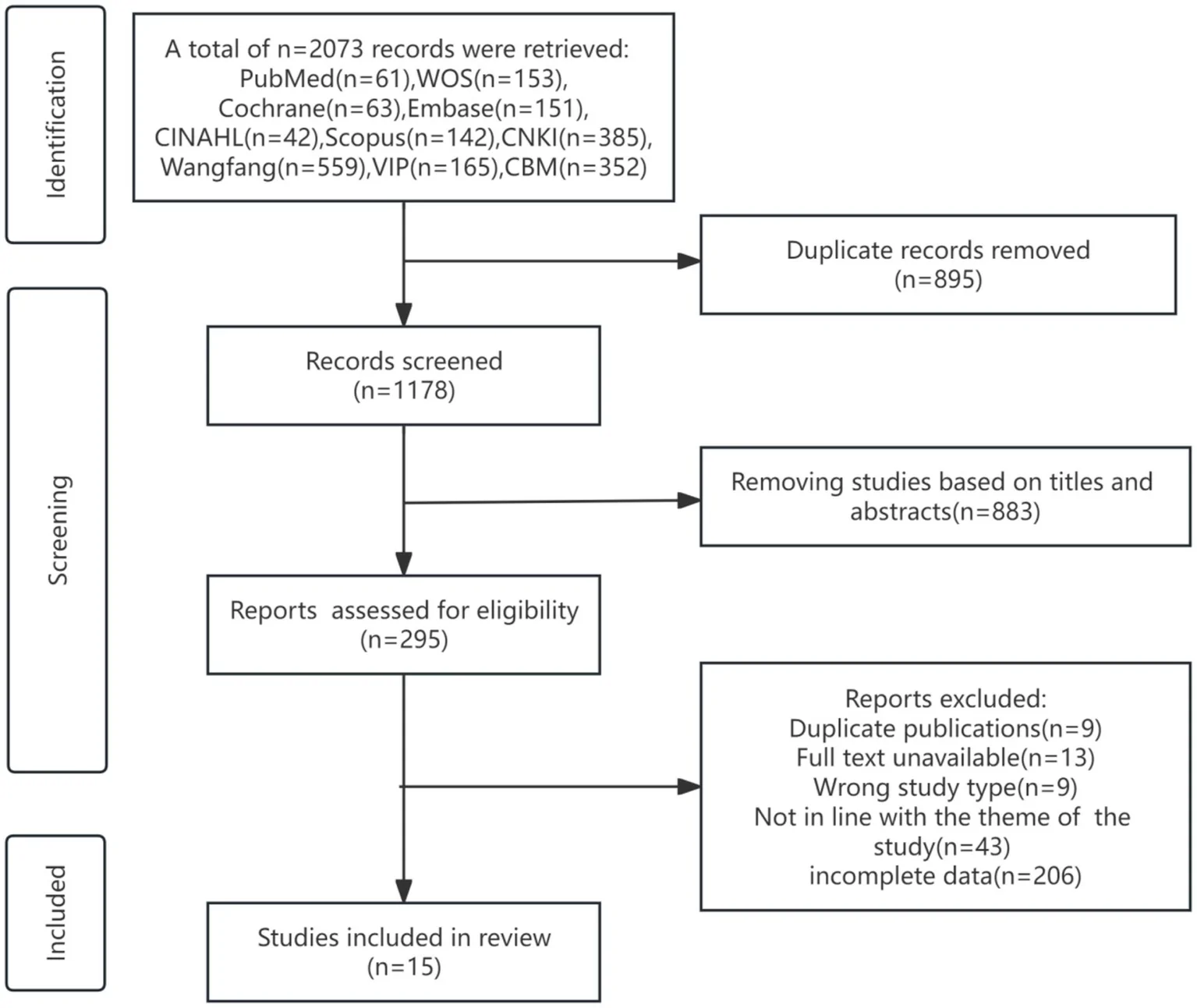
PRISMA flowchart of the selection of studies of self-management interventions for arteriovenous fistulas in hemodialysis patients.

### 2.5 Literature screening and data extraction

The retrieved literature was imported into NoteExpress software for reprocessing. Two trained researchers independently screened the titles and abstracts according to the inclusion and exclusion criteria, and then re-screened the full text to determine the final included literature. Data extraction was performed after a full reading of the full text, and the extracted information was entered into a Microsoft Excel database to create a fully accessible dataset covering authors, study country and publication year, sample size, study design, theoretical foundation, protocol of intervention, intervention time parameters, and outcome indicators. The key intervention protocol-related information included the developers and implementers of the intervention, as well as the specific content and delivery format of the intervention. In the above process, when two researchers disagreed, the third researcher made the judgment.

## 3 Results

### 3.1 Results of literature screening

A total of 2073 articles were retrieved, and 15 articles were included after eliminating duplicates and reviewing titles, abstracts, and full texts (8, 9, 13–25). The literature screening process is shown in Figure 1. Among these, 8 articles were published in English (8, 9, 13, 16–20), while 7 articles were published in Chinese (14, 15, 21–25). 9 articles originated from China (13–15, 19, 21–25), 2 from Turkey (16, 18), 1 from Portugal (9), 1 from Brazil (17), 1 from Korea (8), and 1 from Saudi Arabia (20). The research types comprised 9 randomized controlled trials (13–15, 17, 18, 22–25), 6 quasi-experimental studies (8, 9, 15, 16, 19–21). The basic characteristics of the included studies are shown in Table 1.

**Table 1:** Characteristics of included studies.

Author	Country/Year	Sample size (control/experimental group)	Study design	Theoretical foundation	Protocol of intervention	Intervention time parameters	Outcome indicators
Lee H et al. (8)	Korea/2023	25/25	Quasi-Experimental Study	/	Upper limb strength training is progressively conducted in eight stages. A health information magazine is used to provide patients undergoing hemodialysis with limited-phosphorus diet management, which covers nutritional guidance on phosphorus metabolism, the dangers of high phosphorus, and low-phosphorus food choices. Health education on AVFs vascular management is delivered to patients through videos. An action plan checklist is reviewed weekly to monitor patients’ exercise performance, AVFs self-management, and implementation of the limited-phosphorus diet. Additionally, text messages are sent to provide continuous intervention support.	Once a week, 40 min per session, for a total of 8 weeks	Physiological indicators, Psychological state
Clemente Neves Sousa (9)	Portugal/2021/	48/41	Quasi-Experimental Study	/	Theoretical Training: Conduct six 30-min educational group sessions on “AVFs Care” (≤8 people per group) to teach patients how to recognize situations that may affect the function of their AVFs. Practical Training: Initiated one week after the theoretical training and lasting for two weeks (during the early stages of hemodialysis treatment), the interactive training course develops patients’ skills in inspecting and palpating the AVFs arm.	3 months	Self-management ability, Physical function
Jin LS et al. (13)	China/2024	55/55	Randomized Controlled Trial	/	Preparation: Renovate the science popularization workshop area, produce thematic videos co-performed by medical staff and patients, and establish a WeChat group. Before Class: Upload 5-10-min thematic videos to the WeChat group for patients and their families to learn. The content of the thematic videos includes: fistula assessment, care, dietary management, exercise, and complication management. During Class: Doctors and nurses conduct theoretical knowledge lectures and practical operation demonstrations in the workshop, inviting patients and their families to interact, provide timely feedback, and offer guidance. After Class: Nurses assess the patients’ fistula status and their understanding of the knowledge.	2 times per week, 30–60 min per session, for a total of 4 weeks.	Self-management ability, Patient Experience, Physical function、Quality of life
Lin Ping et al. (14)	China/2024	45/45	Randomized Controlled Trial	/	Preparation Phase: Conduct a survey on patients’ self -care abilities to identify common nursing issues. Produce thematic micro-videos covering topics such as fistula puncture site care, medication considerations, prevention and control of complications, dietary nutrition and dry weight management, interpretation of laboratory test results, and exercise management. Set up the workshop to create an interactive learning environment. Intervention Implementation: Before Class: Push the micro-videos to the WeChat group to guide pre-class learning. During Class: Conduct the intervention following the sequence of video playback+ instructor demonstration (30 min)-feedback education (10 min)-peer education (10 min)-patient interaction (10 min). After Class: Assess the patients’ fistula condition and knowledge retention during hemodialysis. Distribute learning diaries to promote reflection. Praise outstanding learners and organize experience -sharing sessions within the group weekly.	Once a week, 60 min per session, for a total of 6 weeks	Self-management ability, Patient experience, Psychological State, Complication outcome
Tian Yuanru et al. (15)	China/2024	35/35	Randomized Controlled Trial	The PRECEDE-PROCEED Model	Conduct face-to-face communication with patients to identify factors influencing their self-management of AVFs. For predisposing factors: one-on-one skill training, including postoperative protection, dialysis maintenance, and home management. For enabling factors: on the day after surgery, learn basic knowledge of AVFs; at one week post-surgery, assess patients’ adherence to medical advice and answer questions; at four weeks post-surgery, conduct peer education and reinforce treatment adherence. For reinforcing factors: daily supervision via WeChat group, verify patients’ maintenance status, and encourage experience sharing among patients.	1 month	Self-management ability, Complication outcome
Mollaoğlu M et al. (16)	Turkey/2012	32	Quasi-Experimental Study	/	Conduct face-to-face interviews with patients to explain, discuss, and demonstrate the content of the AVFs education manual, including precautions for AVFs, AVFs care, and management of complications.	2 weeks	Self-management ability, Psychological state
Pessoa NRC et al. (17)	Brazil/2024	27/28	Randomized Controlled Trial	Orem’s General Nursing Theory	During the patient’s dialysis process, a 9.7-inch tablet computer was used to play the educational video” AVFs Care” twice. The content covers self-care actions that patients should take before and after the creation of the AVFs and during its use in hemodialysis, as well as the prevention and monitoring of complications. Patients also received the routine verbal education provided by the dialysis center	2 weeks	Self-management ability
Dilbilir Y et al. (18)	Turkey/2023	35/31	Randomized Controlled Trial	/	Once a week, a 30-45-min face-to-face session was conducted in the training room of the hemodialysis unit to explain one topic from the AVFs Care Patient Education Manual. The content included: kidneys, kidney function, chronic kidney disease, etiology, symptoms, staging, types of treatment, hemodialysis treatment, vascular access for hemodialysis, definition and maturation of AVFs, thrill assessment, AVFs care, AVFs complications, and principles for avoiding complications. After the training, the AVFs Care Patient Education Manual was distributed to facilitate daily reference and review.	Once a week, 30–45 min per session, for a total of 4 weeks.	Self-management ability
Chen J et al. (19)	China/2021	89/107	Quasi-Experimental Study	/	Nurses explain the types of AVFs complications and nursing measures to patients and their families after admission; they provide detailed instructions on the dosage and duration of medications, strictly administering drugs according to medical orders. Nurses also tailor their education on the principles of AVFs based on the patients’ comprehension abilities. Sterile procedures are performed before puncture, and the puncture site is regularly cleaned. Nurses dynamically monitor the patients’ vital signs, drain and incise local abscesses, and provide appropriate antibiotic therapy.	/	Self-management ability, Physiological indicator, Psychological state, Patient experience, Complication outcome, Quality of life
Alsaqri SH et al. (20)	Saudi Arabia/2019	71	Quasi-Experimental Study	/	Distribute the educational module materials on AVFs care to patients and provide appropriate explanations. The content of the educational module includes: the meaning of AVFs, the purpose of AVFs, complications of AVFs and their management.	2 weeks	Self-management ability
WANGH et al. (21)	China/2023	90/90	Quasi-Experimental Study	Root Cause Analysis	Establish a fistula quality management team based on the Root Cause Analysis method. Utilize the “Five Whys” technique to trace the causes of AVFs complications, optimize the fistula improvement plan, and standardize the management system for fistulas. In conjunction with the 6S principles, implement a graded management approach for patients’ blood vessels, select appropriate puncture methods, design AVFs warning rings, and conduct real -time monitoring of arm compression to prevent fistula injury.	1 year	Self-management ability, Complication outcome
Liu Shaoyun et al. (22)	China/2024	100/100	Randomized Controlled Trial	The Contractual Management Model	Assess patients to develop individualized nursing intervention plans, including health education on hemodialysis and AVFs care, fistula exercise training, and prevention and treatment of complications; sign contracts, negotiate intervention plans, and supervise and correct atients’ unhealthy behaviors; regularly organize peer support group meetings for case sharing, emotional venting, and encourage family members to monitor patients’ diet and fluid intake, and regularly return to the hospital to review the quality of the fistula and the implementation of the contract.	1 year	Self-management ability, Physiological indicators, Quality of life, Complication outcome
Chen Zexun et al. (23)	China/2021	48/48	Randomized Controlled Trial	The Transtheoretical Model of Behavior Change	Assessment of Patient Stages: Use the Stages of Change Measure and self-designed questions to evaluate the patient’s stage of readiness for self-care. Stage-specific Interventions: Precontemplation Stage: Grouplecture (30 min): Emphasize the importance of self-care. Casevideo (30 min): Demonstrate successful treatment cases. Comparative images (15 min): Show long-term outcomes vs. adverse consequences. Individual counseling: Motivate behavioral change. Contemplation Stage: Address concerns and eliminate doubts about self-care. Establish a support system involving healthcare providers, patients, and family members. Preparation Stage: Provide self-care manuals and conduct face-to-face demonstrations to teach self-care knowledge. Action Stage: Provide feedback on the patient’s self-care effectiveness. Offer positive reinforcement and assessment to guide self-care behaviors. Maintenance Stage: Conduct regular follow-up via phone calls (15 min per call, every two weeks) and home visits (30 min per visit, monthly) to monitor and evaluate self-care practices. Provide psychological counseling and technical support if the patient interrupts self-care behaviors.	6 months	Self-management ability, Physiological indicators, Complication outcome, Patient experience
Pang Menglin et al (24)	China/2022	56/56	Randomized Controlled Trial	Ebbinghaus Forgetting Curve	Multimodal Health Education: Picture-Based Dialogue Education: One learning theme per week, clarifying misconceptions and reinforcing nursing key points through picture and text discussions. Theory of Planned Behavior: Invite successful patients to share their experiences, involve family members in supervision, and distribute health manuals to standardize behaviors. Empowerment Education: Identify existing patient issues, set nursing goals and tasks, and provide regular feedback on patients’ self-care behaviors and capabilities by nurses. Transtheoretical Model: Organize social activities (demonstration teaching+ thematic discussion) to strengthen the cognitive connection between behavior and health. Follow-Up Management: Push protective knowledge through WeChat groups, conduct out-of-hospital follow-ups to answer questions, and assess behavioral adherence.	3 months	Self-management ability
Deng Yanan et al. (25)	China/2019	60/60	Randomized Controlled Trial	The Transtheoretical Model of Behavior Change	Healthcare collaboration established a hemodialysis specialty nursing team to provide a three-stage intervention for patients. During the induction period, the team focused on implementing AVFs health education, personalized psychological counseling, and infection prevention guidance for patients. In the continuous dialysis period, an online community was established to implement remote management and enhance patients’ self-care abilities. In the extended care period, multidimensional follow-up through phone calls, home visits, or digital platforms guided patients in fistula maintenance, dynamically assessed and adjusted the nursing plan, and simultaneously constructed a family support system.	1 year	Self-management ability, Complication outcome

### 3.2 Intervention planners and implementors

Of the 15 studies, 10 reported the full involvement of nurses in the intervention process (13–15, 17–19, 21, 22, 24, 25), of which 7 were designed and implemented by clinicians and nurses together (13–15, 17, 21, 24, 25). The remaining five studies (8, 9, 16, 20, 23) did not explicitly report the occupational background of the participants.

### 3.3 Theoretical foundations

Among the 15 studies included in this review, 7 studies described specific nursing theories, models, or methods for self-management interventions targeting AVF. Both the Transtheoretical Model of Behavior Change (23) and the phased continuous nursing model (25) are based on the stage characteristics of behavior change, and different interventions are implemented for patients at different stages. Among them, Chen et al. (23) evaluated the behavioral intervention needs and behavioral characteristics of patients in each stage according to the five stages of pre-intention, intention, preparation, action and maintenance, and provided phased self-management guidance. Another study implemented staged continuous nursing for patients, which improved the self-care ability of patients through staged assessment and guidance, thus reducing the occurrence of complications (25). The PRECEDE-PROCEED Model will affect the individual factors as tendency, and promote healthy behaviors factors, strengthening factors and the factor analysis as the breakthrough point, accurate formulate improve arteriovenous fistula self-management health education plan, establishing the health behavior (15). The above three studies all regarded patients’ self-management ability as a dynamic process, and implemented adaptive intervention on the basis of phased assessment. Orem’s General Nursing Theory in order to enhance self-care ability as the core framework, through education video improve hemodialysis patient self-management practices of AVF (17). the Contractual Management Model (22): through the signing of contracts, the nurse–patient binding is carried out, the functions, responsibilities and mutual relations of the two sides are clearly agreed, the feedback mechanism is established, and the patients are supervised and guided to conduct the self-management of mobile venous fistula, so as to strengthen the patients’ identity with the intervention goals and improve their compliance. The common point between the two is to emphasize the subjectivity of patients, and enhance the self-management ability of AVF in hemodialysis patients through empowerment, feedback and participation mechanism. Root Cause Analysis (21): this study adopts the method of “five whys” to track the root cause of AVF related adverse events in patients receiving hemodialysis. By identifying management loopholes, locating core problems, and promoting program standardization. Multimodal health education based on Ebbinghaus forgetting curve (24): information reproduction at multiple time nodes combined with the patient’s age, family background, self-report and observer report. Both studies took specific problems as starting points, and the former designed targeted intervention strategies for management loopholes and the latter for knowledge forgetting curve, respectively. The application focus of each theory is different, but they all have the common feature of personalized intervention.

### 3.4 Contents of the intervention

The intervention content of the 15 included studies mainly included the introduction, evaluation, nursing, precautions and complications of AVF (11–14, 17–27). Among them, 4 studies involved diet management (13, 14, 21, 27), 3 studies concerned the treatment of hemodialysis, vascular access and complication prevention (14, 19, 24), and 3 studies involved exercise management (12, 13, 20), and 2 studies provided medication guidance for patients (14, 22). 1 study, respectively, mentioned the anatomy and function of urinary system, chronic kidney disease (19), dry weight management, and interpretation of test results (13).

### 3.5 Forms of intervention

Self-management interventions for AVF in hemodialysis patients can be categorized into 4 forms: (1) Individualized face-to-face education is the most basic intervention form with the widest coverage. In 12 studies (8, 14–16, 18–25) researchers contacted subjects directly to understand their real experience, difficulties and self-management needs of arteriovenous fistula. Provide targeted and personalized interventions. (2) multimodal health education: In 12 studies (8, 9, 13–18, 20, 23–25) this form was adopted. The educational media was gradually expanded from traditional PPT, popular science pictures, and health education manuals to video, animation, and other multimedia forms for popularizing the knowledge of internal fistula nursing. To help patients master the self-detection skills of arteriovenous fistula through practical teaching. (3) Peer support: 6 studies set up peer support links (13–15, 22–24). through patient communication meetings, peer education and other activities, communication platforms were built to promote emotional communication, experience sharing and information exchange between patients. (4) Mobile health management:8 studies (8, 13–15, 22–25) carried out transitional care and follow-up with the help of wechat, public account, short message, telephone and other means. Notable is, most of the research is not in the form of a single, but combinations of multiple interventions. For example, the combination of simultaneous use of individualized face-to-face education, multimodal health education, and mobile health management has been repeated in multiple studies (8, 13–17, 22–25). This combined intervention strategy reflects the complexity of self-management interventions for AVF.

### 3.6 Overall intervention period, frequency, duration of single intervention

7 studies (8, 9, 13, 14, 18, 23, 24) describes in detail of AVF in self-management intervention time parameters, overall intervention period span from 2 weeks to 8 weeks, number of intervention for 1 to 8 times, single intervention duration ranging from 15 min to 60 min. There was a lack of consistency in the selection of intervention duration and frequency among various studies, and no clear evidence-based basis was provided. This heterogeneity reflects the lack of consensus in the field regarding the parameters of intervention.

### 3.7 Outcome indicators

The outcome indicators mainly involved 7 aspects, (1) Self-management ability: All 15 studies involved, The measurement tools used include The Scale of Knowledge, Attitude, and Practice of Self-Care with AVF (17), the Scale of Assessment of Self-Care Behaviors with Arteriovenous Fistula in Hemodialysis (ASBHD-AVF) (9, 14, 15, 18, 22, 24), the Hemodialysis Patients Self-Management Scale (22), the Exercise of Self-Care Agency Scale (ESCA) (19, 24, 25), self-efficacy (8) and self-made scale (13, 20, 21, 24, 25), etc. (2) Physical function: Two studies evaluated cognitive function (9) and motor function (13). (3) Physiological and biochemical indicators: one study focused on the physiological and biochemical indicators of AVF in hemodialysis patients, including upper limb muscle strength, blood flow velocity, blood calcium and phosphorus levels (8). (4) Complication outcomes: 7 studies reported the incidence of complications of arteriovenous fistula (14, 15, 19, 21–23, 25). (5) Psychological state: 4 studies focused on the psychological state of patients, including positive psychological state (14), anxiety (16, 19), depression (19), etc. (6) Quality of life: 3 studies evaluated the quality of life of patients, including kidney disease questionnaire (KDQ) (22), The World Health Organization Quality of Life Scale(WHOQOL-BREF) (19) and the 12-Item Short Form Health Survey (13). (7) Patient experience: 5 articles reported compliance (14, 19) or satisfaction (13, 19, 23). In general, the choice of outcome measures varied greatly among the studies, and the measurement tools used were also different, with most studies relying on subjective measures reported by patients.

## 4 Discussion

This scope review aims to systematically review the theoretical foundation, Intervention planners and implementors, intervention content, intervention form, overall intervention period, frequency and duration of single interventionand, outcome indicators of self-management intervention programs for AVF in hemodialysis patients. The 15 included studies showed that this field had formed a certain research foundation, and the theoretical application had gradually attracted attention, although the evidence was still insufficient. Nurses were the main participants in the formulation and implementation of intervention programs, and the intervention form had gradually expanded from traditional individualized face-to-face education to peer support, with the help of wechat platform, telephone, SMS and other Internet platforms. There was significant differences among the studies in the design of intervention content, overall intervention period, frequency and duration of single intervention, and outcome indicators. In report intervention plan formulation and implementer of 10 studies (13–15, 17–19, 21, 22, 24, 25), plan how to formulate jointly by clinical doctors and nurses, and implementer is given priority to with the nurse. This finding is consistent with clinical practice. Hemodialysis patients need long-term and regular treatment. Nurses have frequent contact with patients during dialysis, which makes it easier to observe the use of AVF, identify the existing self-management problems of patients, and guide patients to adjust their behavior through intervention. AVF function, however, to maintain the same by vascular conditions, piercing technology, blood pressure control, nutritional status, the phosphorus, blood glucose and blood lipid levels, mental state and family support and so on many factors. KDOQI guidelines emphasize 2019 vascular access should by multidisciplinary practitioners involved in vascular access management, combined with the patients individual life to plan the whole management (1). However, in the studies included in this review, there are few interventions involving multidisciplinary teams, the existing programs are nurse-led, and the intervention content has not yet formed a unified standard, mainly focusing on the assessment, nursing, precautions and related knowledge of AVF complications (8, 9, 13–25). It is less involved in urinary system and chronic kidney disease (18), hemodialysis (15, 18, 22), diet management (24, 9–14–15), medication guidance (14, 19), exercise management (8, 13, 14), and so on. These deficiencies suggest that it is necessary to introduce dietitians, rehabilitation therapists, psychological counselors and other multidisciplinary professionals to participate in the formulation of future intervention programs.

Seven of the included studies mentioned the nursing theory, model or method used in the intervention program, which showed that researchers had paid attention to the guiding role of theoretical basis in the construction of the program. From the existing research, it is found that the emphasis of each theory is different. The Transtheoretical Model of Behavior Change (23, 25) is helpful to explain the stages of patients’ behavior change. The PRECEDE-PROCEED Model (15) helps to identify behavioral influencing factors from three levels: predisposing factors, facilitating factors and reinforcing factors. Orem’s General Nursing Theory (17) emphasizes the gap between patients’ self-care needs and ability; the Contractual Management Model (22) emphasizes the agreement of the common goals of nurses and patients and the allocation of responsibility; the Ebinhaus forgetting curve (24) provides the time rhythm basis for the repeated reinforcement of health education. These theories, models and methods provide different entry points for self-management intervention of AVF, but it is worth noting that most of these studies mentioned the name of the theory in the program design, and few specified how the theory guided the design of intervention content, implementation process or effect evaluation, which weakened the interpretability and replicability of the program to a certain extent. The value of a theory, model, or method lies not only in defining the theoretical basis of an intervention, but also in providing a systematic path for program development and implementation. For example, Yang et al. constructed a transitional intervention for adolescents with chronic kidney disease based on the integrated health behavior change theory, and transformed “knowledge and belief”, “self-regulation skills” and “social promotion” into specific measures such as individualized health education, goal setting and self-monitoring, regular feedback and nurse-family support, respectively. Outcome indicators such as transition readiness, medication adherence and self-efficacy were selected accordingly, reflecting the role of the theoretical framework in establishing the logical chain of “behavioral influencing factors, intervention strategies, and evaluation indicators” (26). The hemodialysis self-management intervention developed by Gilva et al. is based on the psychological theory of behavior change. The strategies of problem solving, goal setting, action plan, progress feedback and relapse prevention are embedded in the management of fluid, diet, medication and exercise. It is shown that the theory can help researchers transform abstract self-management goals into intervention programs with clear behavioral targets, implementation steps and reinforcement mechanisms (27). The UK Medical Research Council (MRC) Framework for Complex intervention Research also emphasizes that the interaction between project theory and implementation context should be continuously considered in the stages of intervention development, feasibility evaluation, effect evaluation and implementation (28). Future research should further clarify the corresponding relationship between the theoretical framework and each link of the intervention, so that the theoretical guidance can truly run through the whole process of program construction, rather than just staying at the concept level.

The intervention forms of the included studies showed obvious combinatorial characteristics. Individualized face-to-face education (12 articles) (8, 14–16, 18–25), multimodal health education (12 articles) (8, 9, 13–18, 20, 23–25) and mobile health management (8 articles) (8, 13–15, 22–25) were the three most frequently reported forms, and the vast majority of studies used two or more interventions simultaneously The way. Peer support was used in 6 studies (13–15, 22–24). Self-management of AVF involves knowledge mastery, behavioral training and long-term follow-up. A single intervention form is difficult to cover all needs, so a combination of forms has become a common choice. However, obvious differences between the research on the intervention of the concrete implementation. The content of educational materials, the functional design and interaction frequency of mhealth tools, and the follow-up period are different, and the organization of peer support is also lack of unified norms. Some studies have found that e-health intervention may help to improve the quality of life and treatment compliance of hemodialysis patients, and reduce anxiety and depression, while also acknowledging that there is a large heterogeneity in the included studies (29). A systematic review and meta-analysis of maintenance hemodialysis patients reported similar findings: nurse-led peer support improved self-management, quality of life, and depressive symptoms, but the standardization of the intervention program was limited (30). This discrepancy makes it difficult to directly compare the results of the various studies and makes it more difficult to choose the optimal regimen in clinical practice. Follow-up studies should be clear all kinds of intervention in the form of core elements and the implementation of standards, at the same time explore the optimal configuration of intervention form combination, clear the different forms in the knowledge, behavior change, emotional support and other relative contribution of target and synergy.

This review found that the overall intervention period in the reported studies ranged from 2 weeks to 8 weeks, and the intervention frequency and the duration of a single intervention varied greatly (8, 9, 13, 14, 18, 23, 24). This indicates that there is no consensus on the dosage of self-management intervention for AVF at present. Short-term education may help to improve patients’ self-management ability of AVF, but self-management of AVF requires long-term adherence. Whether short-term intervention can support patients to form stable habits needs to be further explored. At present, only three studies (21, 22, 25) have conducted a 1-year follow-up. Most of the studies were followed up within 3 months after the end of the intervention, but the maintenance of self-management behaviors in hemodialysis patients faces special challenges, such as age-related changes in cognitive function (31), the risk of cerebral hypoxia caused by anemia (32), and the possible relaxation and weakening of awareness caused by long-term dialysis, which may weaken the long-term consolidation of behaviors. Therefore, future research needs to pay more attention to the design of follow-up cycle, explore the nodes of intervention reinforcement, and pay attention to the trajectory of patients’ self-management behavior over time.

The included studies took the self-management ability of AVF in hemodialysis patients as the main outcome index, which directly reflected the changes in the self-management ability of AVF in hemodialysis patients. However, the scales used in different studies are not completely consistent, and some studies use self-made scales, and the reliability and validity of the scales are not enough to report, which affects the comparability of the results. In contrast, physiological and biochemical indexes, complications, psychological state, physical function and quality of life and patients experience fewer reports. Only one study (8) focused on physiological and biochemical indicators such as upper limb muscle strength, blood flow rate, blood calcium and blood phosphorus. Seven studies (14, 15, 19, 21–23, 25) reported fistula complications. Important patient outcomes such as psychological status, physical function, quality of life, and patient experience also had relatively limited coverage. This suggests that the current research focuses more on “whether patients master the knowledge and skills of self-management of AVF,” but there is no consensus on whether these behavioral changes are related to the long-term use of arteriovenous fistula and patients’ life experience. SONG - HD consensus pointed out that the function of vascular access should be as an important core of hemodialysis vascular access research outcome, whether in the study should focus on vascular access in the case of no additional intervention continued usage, and patients can complete prescription dialysis (1). Therefore, future studies can gradually pay attention to clinical outcomes such as the number of vascular access interventions and the incidence of vascular access complications on the basis of retaining proximal outcomes such as self-management ability. At the same time, more attention should be paid to patient-reported outcomes, such as pain, anxiety, quality of life, and patient experience, so as to build a comprehensive evaluation system that takes into account self-management behavior, clinical function and patient experience.

## 5 Limitations

Our study has several limitations. First, the literature search was limited to Chinese and English and was implemented through the database language filter in the retrieval stage. This choice was based on the language ability of the research team to ensure the accuracy of data extraction and analysis, but relevant studies in other languages may be missed. Second, gray literature was not included. Considering that the retrieval channels of gray literature were scattered, not peer-reviewed, and with uneven quality, there was a certain risk of publication bias. Third, this study only focused on the self-management intervention of arteriovenous fistula in maintenance hemodialysis patients, and did not include related studies of patients in the perioperative period and other stages, so it could not fully reflect the full picture of evidence for the whole course management of arteriovenous fistula.

## 6 Conclusion

The scope of this review include 15 study, the system describes the hemodialysis patients with arteriovenous fistula in existing evidence map, self-management intervention from intervention programme planners and implementers, theoretical foundation, content, the form of intervention, overall intervention period, frequency and duration of single intervention, and outcome indicators six dimensions presented the overall characteristics of the field. In terms of intervention planners and implementers and interventions, the existing implementation mainly dominated by the nurse intervention, use individualized face-to-face education, multimodal health education, peer support and mobile health management, and other combinations. Some studies use the theoretical foundation such as the Transtheoretical Model of Behavior Change, Orem’s General Nursing Theory, The PRECEDE-PROCEED Model, the Contractual Management Model, Root Cause Analysis to guide intervention design, but the corresponding relationship between theory and specific intervention content and outcome indicators is not clear, and the theory stays at the level of program design, rather than the guidance framework throughout the whole process of intervention. The content of intervention mainly focused on the assessment, nursing and related knowledge of AVF complications, and less involved in diet management, medication guidance, exercise management and psychological support. The overall duration of reported study interventions ranged from 2 to 8 weeks, the frequency ranged from 1 to 8 sessions, and the duration of a single session ranged from 15 min to 60 min. However, most of the studies did not adequately report the temporal characteristics of the intervention, and the reasonable interval of intervention intensity has yet to be determined. In terms of outcome indicators, existing studies mainly focus on patients’ self-management ability, and reports on physiological and biochemical indicators, physical function, psychological state, complications, quality of life and patient experience are relatively limited. In general, the inadequate applications face theory, intervention with limited degree of standardization, loss of multidisciplinary collaboration, outcome indicators is not unified, and long-term follow-up problems such as lack of evidence. While clarifying the theoretical basis and core elements of intervention, future research should expand the intervention model of multidisciplinary collaboration, standardize the reporting of intervention period, frequency and duration of single intervention, incorporate more clinical and patient-reported outcomes on the basis of proximal outcomes such as self-management ability, and construct a more complete evaluation system.

## Data Availability

The datasets used and/or analyzed during the current study are available from the corresponding author on reasonable request.

## Footnotes

1
https://osfio/4wfzm
