# Supplementary material for: Scope review of self-management intervention for arteriovenous fistula in hemodialysis patients
Source: Front Public Health. 2026 Sep 15;14:1859375. doi: 10.3389/fpubh.2026.1859375 (PMC13619570; doi:10.3389/fpubh.2026.1859375)
Supplement: Supplementary file 1 [file Data_Sheet_1.pdf]

The search strategy for the PubMed database (61)

No	**Search History**
#1	renal dialysis[MeSH Terms]
#2	(renal dialysis[Title/Abstract] OR Dialyses, Renal [Title/Abstract] OR Renal Dialyses[Title/Abstract] OR Dialysis,Renal[Title/Abstract]OR Hemodialysis [Title/Abstract]OR Hemodialyses [Title/Abstract]OR Dialysis,Extracorporeal [Title/Abstract]OR Dialyses, Extracorporeal [Title/Abstract] OR Extracorporeal Dialyses[Title/Abstract]OR Extracorporeal Dialysis[Title/Abstract] OR maintenance hemodialysis[Title/Abstract])
#3	#1 OR #2
#4	Arteriovenous Fistula[MeSH Terms]
#5	(Arteriovenous Fistula[Title/Abstract] OR Fistula, Arteriovenous[Title/Abstract] OR Fistulas, Arteriovenous[Title/Abstract] OR Arteriovenous Fistulas [Title/Abstract] OR vascular fistula [Title/Abstract] OR vascular access [Title/Abstract] OR vascular pathway[Title/Abstract])
#6	#4 OR #5
#7	self management[MeSH Terms]
#8	(self-management[Title/Abstract] OR Self Management[Title/Abstract] OR Management Self[Title/Abstract] OR Self care[Title/Abstract] OR Care, Self[Title/Abstract] OR Self-Care[Title/Abstract] OR Self-Control [Title/Abstract] OR Self Regulation[Title/Abstract] OR Regulation, Self [Title/Abstract] OR Self-Regulation[Title/Abstract] OR Self Control [Title/Abstract] OR Control, Self[Title/Abstract])
#9	#7 OR #8
#10	(#1 OR #2) AND (#4 OR #5) AND (#7 OR #8)

The search strategy for the Web of Science (153)

**No**	**Search History**
#1	TS=(renal dialysis OR Dialyses, Renal OR Renal Dialyses OR Dialysis, Renal OR Hemodialysis OR Hemodialyses OR Dialysis, Extracorporeal OR Dialyses, Extracorporeal OR Extracorporeal Dialyses OR Extracorporeal Dialysis OR maintenance hemodialysis)
#2	TS=(Arteriovenous Fistula OR Fistula, Arteriovenous OR Fistulas, Arteriovenous OR Arteriovenous Fistulas OR vascular pathway OR vascular fistula OR vascular access)
#3	TS=(self-management OR Self Management OR Management Self OR Self care OR Care, Self OR Self-Care OR Self-Control OR Self Regulation OR Regulation, Self OR Self-Regulation OR Self Control OR Control, Self)
#4	#1 AND #2 AND #3

The search strategy for the the Cochrane database (63)

No	Search History
#1	MeSH descriptor:[Arteriovenous Fistula]explode alltrees
#2	(Ateriovenous Fistula OR Fistula,Ateriovenous OR Fistulas.Ateriovenous OR Ateriovenous Fistulas OR vascular fistula OR vascularaccess OR vascular pathway):ti,ab,kw
#3	MeSH descriptor:[Self-Management]explode all trees
#4	(sel-management OR Self Management OR Management Sel OR Self care OR Care,SelfOR Self-Care OR Selt Control OR Self Regulation OR Regulation, S Self OR Self-Regulation OR Self Control OR Control,Self):ti,ab,kw
#5	MeSH descriptor:[Renal Dialysis]explode alltrees
#6	(renal dialysis OR Dialyses,Renal OR RenalDialyses OR Dialysis,Renal OR Hemodialysis OR Hemodiayses OR Dialysis,Extracorporeal OR Dialyses, Extracorporeal OR Extracorporeal Dialyses OR Extracorporeal Dialysis OR maintenance hemodialysis):ti,ab,kw
#7	#1 OR #2
#8	#3 OR #4
#9	#5 OR #6
#10	#7 AND #8 AND #9

The search strategy for the EMBASE database (151)

No	Search History
#1	('renal dialysis'exp OR 'renal dialysis' OR 'dialyses, renal' OR 'renal dialyses' OR'dialysis, renal' OR 'hemodialysis'/exp OR 'hemodialysis' OR 'hemodialyses' OR 'dialysis, extracorporeal' OR'dialyses, extracorporeal' OR'extracorporeal dialyses' OR'extracorporeal dialysis'/exp OR'extracorporeal dialysis' OR'maintenance hemodialysis'/exp OR 'maintenance hemodialysis')
#2	('arteriovenous fistula'/exp OR'arteriovenous fistula' OR 'fistula, arteriovenous'exp OR 'fistula, arteriovenous' OR 'fistulas, arteriovenous' OR'arteriovenous fistulas'/exp OR'arteriovenous fistulas' OR 'vascular fistula'/exp OR'vascular fistula' OR 'vascular access'/exp OR 'vascular access' OR'vascular pathway)
#3	'self-management'/exp OR'self-management' OR'self management'/exp OR'self management' OR'management self OR'self care'/exp OR'self care' OR'care, self'' OR'self-care'/exp OR'self care' OR'self-control'/exp OR self control'OR 'self regulation'/exp OR 'self regulation' OR'regulation self'/OR'self-regulation'/exp 'self-regulation' OR'self control'/exp OR'self control' OR'control, self
#4	#1 AND #2 AND #3

The search strategy for the CINAHL database (42)

No	Search History
#1	TI(renal dialysis OR Dialyses, Renal OR Renal Dialyses OR Dialysis, Renal OR Hemodialysis OR Hemodialyses OR Dialysis, Extracorporeal OR Dialyses, Extracorporeal OR Extracorporeal Dialyses OR Extracorporeal Dialysis OR maintenance hemodialysis)OR AB(renal dialysis OR Dialyses, Renal OR Renal Dialyses OR Dialysis, Renal OR Hemodialysis OR Hemodialyses OR Dialysis, Extracorporeal OR Dialyses, Extracorporeal OR Extracorporeal Dialyses OR Extracorporeal Dialysis OR maintenance hemodialysis)
#2	TI(Arteriovenous Fistula OR Fistula, Arteriovenous OR Fistulas, Arteriovenous OR Arteriovenous Fistulas OR vascular fistula OR vascular access OR vascular pathway) OR AB(Arteriovenous Fistula OR Fistula, Arteriovenous OR Fistulas, Arteriovenous OR Arteriovenous Fistulas OR vascular fistula OR vascular access OR vascular pathway)
#3	TI(self-management OR Self Management OR Management Self OR Self care OR Care, Self OR Self-Care OR Self-Control OR Self Regulation OR Regulation, Self OR self-Regulation OR self Control OR Control, Self)OR AB(self-management OR self Management OR Management self OR self care OR Care, self OR self-Care OR self Control OR Self Regulation OR Regulation, Self OR self-Regulation OR self Control OR Control, Self)
#4	#1 AND #2 AND #3

The search strategy for the Scopus database (142)

No	Search History
#1	TITLE-ABS-KEY ("self-management" OR "Self Management" OR "Management Self" OR "Self care" OR "Care, Self" OR "Self-Care" OR "Self-Control" OR "Self Regulation" OR "Regulation, Self" OR "Self-Regulation" OR "Self Control" OR "Control, Self")
#2	(TITLE-ABS-KEY ("renal dialysis" OR "Dialyses, Renal" OR "Renal Dialyses" OR "Dialysis, Renal" OR "Hemodialysis" OR "Hemodialyses" OR "Dialysis, Extracorporeal" OR "Dialyses, Extracorporeal" OR "Extracorporeal Dialyses" OR "Extracorporeal Dialysis" OR "maintenance hemodialysis")
#3	(TITLE-ABS-KEY ("Arteriovenous Fistula" OR "Fistula, Arteriovenous" OR "Fistulas, Arteriovenous" OR "Arteriovenous Fistulas" OR " vascular fistula" OR "vascular access" OR "vascular pathway")
#4	#1 AND #2 AND #3

The search strategy for the CNKI database (385)

No	Search History
#1	SU=(血液透析+维持性血液透析+肾透析+透析)
#2	SU=(自我管理+自我管理+自我保健+自我控制)
#3	SU=(动静脉内瘘+自体动静脉内瘘+内瘘+血管通路)
#4	#1 AND #2 AND #3

The search strategy for the WanFang database (559)

No	Search History
#1	主题:("血液透析" or "维持性血液透析" or "肾透析" or "透析")
#2	主题:("自我管理" or "自我管理" or "自我保健" or "自我控制")
#3	主题:("动静脉内瘘" or "自体动静脉内瘘" or "内瘘" or "血管通路")
#4	#1 AND #2 AND #3

The search strategy for theVIP database (165)

No	Search History
#1	M=(血液透析+维持性血液透析+肾透析+透析)
#2	M=(自我管理+自我管理+自我保健+自我控制)
#3	M=(动静脉内瘘+自体动静脉内瘘+内瘘+血管通路)
#4	#1 AND #2 AND #3

The search strategy for the CBM database (352)

No	Search History
#1	常用字段=(血液透析 OR 维持性血液透析 OR 肾透析 OR 透析)
#2	常用字段=(自我管理 OR 自我管理 OR 自我保健 OR 自我控制)
#3	常用字段=(动静脉内瘘运动 OR 自体动静脉内瘘 OR 内瘘 OR 血管通路)
#4	#1 AND #2 AND #3
